# Phylogenomic Analyses of Nucleotide-Sugar Biosynthetic and Interconverting Enzymes Illuminate Cell Wall Composition in Fungi

**DOI:** 10.1128/mBio.03540-20

**Published:** 2021-04-13

**Authors:** Julian Schwerdt, Hao Qiu, Neil Shirley, Alan Little, Vincent Bulone

**Affiliations:** a ARC Centre of Excellence in Plant Cell Walls, School of Agriculture, Food and Wine, University of Adelaide, Waite Campus, Glen Osmond, Australia; b Division of Glycoscience, Department of Chemistry, School of Engineering Sciences in Chemistry, Biotechnology and Health, KTH Royal Institute of Technology, AlbaNova University Centre, Stockholm, Sweden; Universidade de Sao Paulo

**Keywords:** biosynthesis, carbohydrates, evolution, fungal cell wall, nucleotide-sugar biosynthesis, phylogenomic analysis

## Abstract

The fungi are an enormously successful eukaryotic lineage that has colonized every aerobic habitat on Earth. This spectacular expansion is reflected in the dynamism and diversity of the fungal cell wall, a matrix of polysaccharides and glycoproteins pivotal to fungal life history strategies and a major target in the development of antifungal compounds. Cell wall polysaccharides are typically synthesized by Leloir glycosyltransferases, enzymes that are notoriously difficult to characterize, but their nucleotide-sugar substrates are well known and provide the opportunity to inspect the monosaccharides available for incorporation into cell wall polysaccharides and glycoproteins. In this work, we have used phylogenomic analyses of the enzymatic pathways that synthesize and interconvert nucleotide-sugars to predict potential cell wall monosaccharide composition across 491 fungal taxa. The results show a complex evolutionary history of these cell wall enzyme pathways and, by association, of the fungal cell wall. In particular, we see a significant reduction in monosaccharide diversity during fungal evolution, most notably in the colonization of terrestrial habitats. However, monosaccharide distribution is also shown to be varied across later-diverging fungal lineages.

## Abstract (precis)

This study provides new insights into the complex evolutionary history of the fungal cell wall. We analyzed fungal enzymes that convert sugars acquired from the environment into the diverse sugars that make up the fundamental building blocks of the cell wall.

## INTRODUCTION

Fungi comprise a kingdom of heterotrophic eukaryotes that have colonized every aerobic habitat on Earth. In doing so, fungi have evolved spectacular morphological, metabolic, and ecological diversity, including the morphologically simplified cytoparasitic Microsporidia, specialist inhabitants of the oceanic igneous crust (*Exophiala*) (1), vascular plant root mutualistic symbionts (mycorrhiza), extinct tree-like *Prototaxites* spp. (2), and even species that have colonized anaerobic deep-sea sediments (3).

The breadth of fungal life history strategies is reflected in the variety and dynamism of the fungal cell wall, a complex matrix of polysaccharides and glycoproteins that forms a protective barrier, facilitates cell adhesion, and is pivotal to morphogenesis. Significant cell wall structural variation evolved among species and morphotypes as fungi adapted to diverse ecological niches (4–6). For example, the cell walls of pathogenic fungi can withstand significant turgor pressure on the infection apparatus by cross-linking melanin to polysaccharides (7). The fungal cell wall remodels itself in response to shifting environmental conditions. For instance, during infection of the human lung by Aspergillus fumigatus, a significant increase in β-glucan abundance is observed in response to the induced hypoxic microenvironment (8). This dynamism is particularly evident in the evasion of host immune responses through cell wall reorganization to mask epitope polysaccharides, which are major targets in the development of antifungal compounds (4, 5, 9).

Interest in characterizing fungal cell wall polysaccharides stems from the dual role of fungi as supporters of ecosystem function (by decomposing biopolymers and other molecules from dead organisms) and as pathogens that cause significant economic damage. Fungi account for the majority of characterized plant diseases (10). Notable examples include Magnaporthe oryzae, the causative agent of the devastating rice blast (11), and Ophiostoma novo-ulmi, which has killed millions of elm trees across the Northern Hemisphere (12). However, since the biochemical characterization of cell walls from hundreds of fungal species from different taxa is laborious and not realistically achievable, *in silico* analyses that inspect enzyme pathways implicated in wall biosynthesis are promising high-throughput alternative methods.

**FIG 1:**
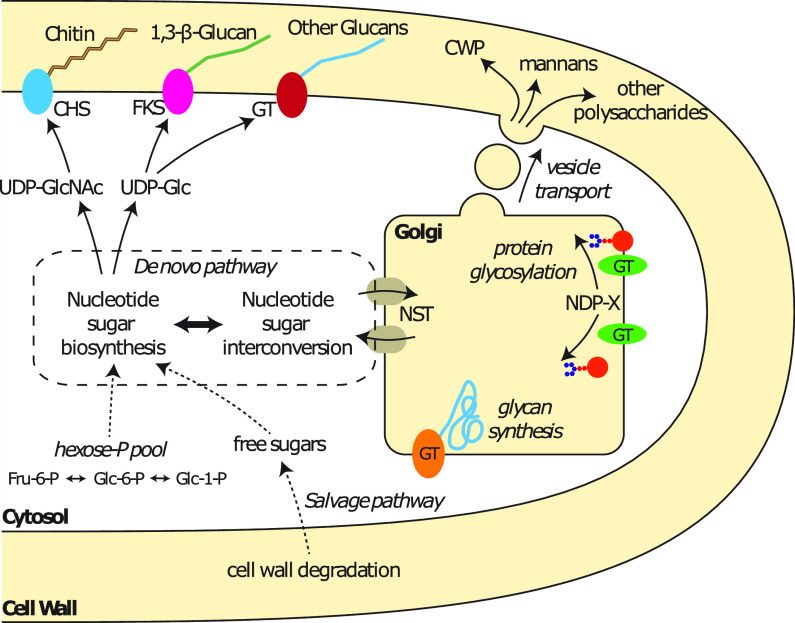
Schematic illustration of cell wall biosynthesis. Chitin and 1,3-β-glucan polysaccharides are synthesized at the plasma membrane by chitin synthase (CHS) and 1,3-β-glucan synthase (FKS), respectively. The glycosyltransferases (GT) responsible for the synthesis of other glucans, such as 1,6-β-glucan, are currently unknown. Cell wall proteins (CWP), mannans, and other polysaccharides are synthesized in the Golgi apparatus and secreted into the cell wall space. Nucleotide-sugars are synthesized and interconverted through the *de novo* pathway into different activated substrates of the various GT. UDP-GlcNAc, UDP-*N*-acetylglucosamine; UDP-Glc, UDP-glucose; NST, nucleotide-sugar transporter.

Glucan, mannan, and chitin are the primary cell wall polysaccharides of biochemically characterized fungi (13). Polysaccharide and glycoprotein biosynthesis is mediated by glycosyltransferases (GT) and takes place either directly at the plasma membrane or in the Golgi apparatus, from which the glycans and glycoconjugates are delivered to the cell wall through secretion vesicles (Fig. 1). Leloir GT are typically membrane-bound proteins that use nucleotide-sugars as substrates. During catalysis, a monosaccharide is transferred to an acceptor, and a phosphate leaving group is simultaneously released (14). The acceptor substrate is often another carbohydrate moiety, and the formation of a glycosidic bond to the nonreducing end of the elongating glycan is catalyzed. As of this writing, 106 GT families are recognized, but a comprehensive understanding of the functional association between polysaccharide and enzyme is hindered by the difficulty in working with membrane-bound proteins and the high level of diversification that many GT families have undergone (15).

However, the biochemical pathways responsible for nucleotide-sugar formation and interconversion are well resolved. Approximately 70 individual nucleotide-sugars have been identified (16). The majority of this diversity is contained within prokaryotes. In fungi, three nucleotide-sugars are responsible for the biosynthesis of the principal cell wall polysaccharides and glycoproteins. These are UDP-glucose (UDP-Glc), utilized by glucan synthases such as FKS for the synthesis of 1,3-β-glucans; UDP-*N-*acetylglucosamine (UDP-GlcNAc), used by chitin synthases for the synthesis of chitin; and GDP-mannose (GDP-Man), the substrate of multiple Golgi mannosyltransferases involved in protein glycosylation and the synthesis of various mannans (Fig. 1).

**FIG 2:**
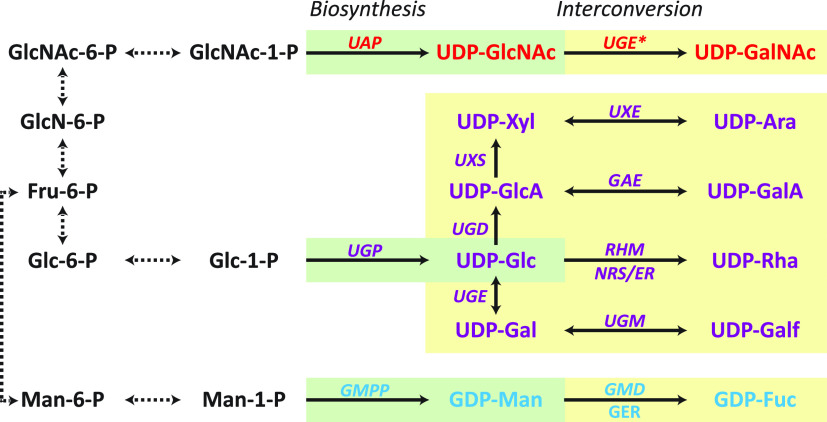
*De novo* nucleotide-sugar biosynthesis and interconversion pathway in fungi. Abbreviations: GDP-Fuc, GDP-fucose; GDP-Man, GDP-mannose; GER, GDP-fucose synthase; GMD, GDP-mannose 4,6-dehydratase; GMPP, mannose-1-phosphate guanylyltransferase; NRS/ER, 3,5-epimerase/4-reductase; RHM, UDP-Glc 4,6-dehydratase; UAP, UDP-GlcNAc pyrophosphorylase; UDP-Ara, UDP-arabinose; UDP-Gal, UDP-galactose; UDP-GalA, UDP-galacturonic acid; UDP-Gal*f*, UDP-galactofuranose; UDP-GalNAc, UDP-*N*-acetylgalactosamine; UDP-Glc, UDP-glucose; UDP-GlcA, UDP-glucuronic acid; UDP-GlcNAc, UDP-*N*-acetylglucosamine; UDP-Rha, UDP-rhamnose; UDP-Xyl, UDP-xylose; UGD, UDP-Glc 6-dehydrogenase; UGE, UDP-Glc 4-epimerase; UGE*, UDP-Glc 4-epimerase/UDP-GlcNAc 4-epimerase; GAE, UDP-GlcA 4-epimerase; UGM, UDP-galactopyranose mutase; UGP, UDP-Glc pyrophosphorylase; UXE, UDP-Xyl epimerase; UXS, UDP-Xyl synthase.

Nucleotide-sugars comprise a nucleoside and two phosphate groups linked to, for instance, a hexose, as in UDP-Glc, UDP-galactose (UDP-Gal), and GDP-Man; a 6-deoxy hexose, as in GDP-fucose (GDP-Fuc) and UDP-rhamnose (UDP-Rha); a pentose, as in UDP-xylose (UDP-Xyl); a hexuronic acid, as in UDP-glucuronic acid (UDP-GlcA) and UDP-galacturonic acid (UDP-GalA); or an amino sugar, as in UDP-GlcNAc and UDP-*N*-acetylgalactosamine (UDP-GalNAc) (17). The interconversion pathway of well-characterized nucleotide-sugars is illustrated in Fig. 2. To date, there has not been a kingdom-wide survey of the nucleotide-sugar interconversion (NSI) pathway in fungi. There is value in carrying out such a survey, for we can identify the nucleotide-sugars available as substrates for GT in each species and inspect how the distribution of activated monosaccharide substrates and the corresponding glycans has changed throughout fungal evolution.

The taxonomy of the kingdom Fungi is incompletely resolved (18); however, consensus exists for a split between flagellated early-branching Chytridiomycota, Neocallimastigomycota, and Blastocladiomycota; between a loose grouping of clades that diversified after losing the flagella (Mucoromycota, Zoopagomycota, Glomeromycota); and between the late-branching Dikarya, composed of the Ascomycota and Basidiomycota (18, 19). The Dikarya are distinct by the independent evolution of multicellular lineages with differentiated tissues and the eponymous presence of binucleate cells that have not undergone karyogamy (20). Microsporidia and *Rozella* spp. synthesize cell walls; however, not until after the Blastocladiomycota diverge is the cell wall present in all stages of the fungal life cycle (19).

The work presented here inspects the presence or absence of nucleotide-sugar biosynthetic and NSI enzymes and the corresponding pathways across 491 fully sequenced taxa spanning all major recognized fungal lineages. The data are used to predict species that have the ability to incorporate specific monosaccharides into their cell walls and illuminate our understanding of how the fungal cell wall has diversified through time.

## RESULTS

### Nucleotide-sugar biosynthesis: pyrophosphorylase phylogeny.

**FIG 3:**
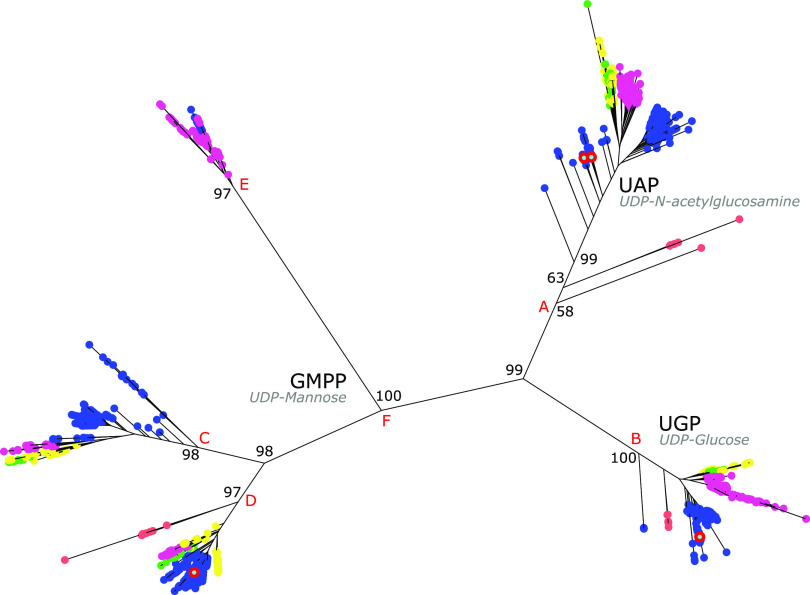
IQ-TREE phylogeny of nucleotide-sugar pyrophosphorylases in 491 fungal taxa. Bootstrap support values are indicated on branches. Nodes are color-coded to represent taxa as follows: blue, Ascomycota; pink, Basidiomycota; green, Blastocladiomycota, Chytridiomycota, and Cryptomycota; yellow, Mucoromycotina, Entomophthoromycotina, Zoopagomycotina, and Kickxellomycotina; orange, Microsporidia. A red circle surrounding a node indicates that the corresponding enzymes or enzyme products have been characterized. For clarity in discussion, major nodes are labeled alphabetically.

As shown in Fig. 3, the IQ-TREE (21) phylogeny for the nucleotide-sugar pyrophosphorylase family resolved four well-supported major clades (A, B, C, and D) that had representatives from all major fungal lineages included in these data, specifically the earliest-branching Chytridiomycota, Neocallimastigomycota, Blastocladiomycota, and Opisthosporidia; the Zoopagomycota and Mucoromycota; and, from the Dikarya, Basidiomycota and Ascomycota. By use of experimentally characterized sequences in the data, biochemical functions were assigned to a single clade composed of UDP-GlcNAc pyrophosphorylases (UAP; split A), which catalyzes the generation of UDP-GlcNAc from UTP and GlcNAc-1-phosphate (GlcNAc-1-P) (Fig. 2); a single clade of UDP-Glc pyrophosphorylases (UGP; split B), which catalyzes the formation of UDP-Glc from UTP and Glc-1-P (Fig. 2); and a single clade of mannose-1-phosphate guanylyltransferases (GMPP; split D), which catalyzes the synthesis of GDP-Man from GTP and Man-1-P (Fig. 2). Sequence homology and protein profile analyses confirmed these assignments and expanded GMPP to include the sister clade to the experimentally verified group (split C) and the sequences following split E. This clade does not have early-branching fungal representatives and is separated from the other GMPP members by a long molecular branch.

### Nucleotide-sugar interconversion: epimerase/dehydrogenase/dehydratase/mutase phylogeny.

**FIG 4:**
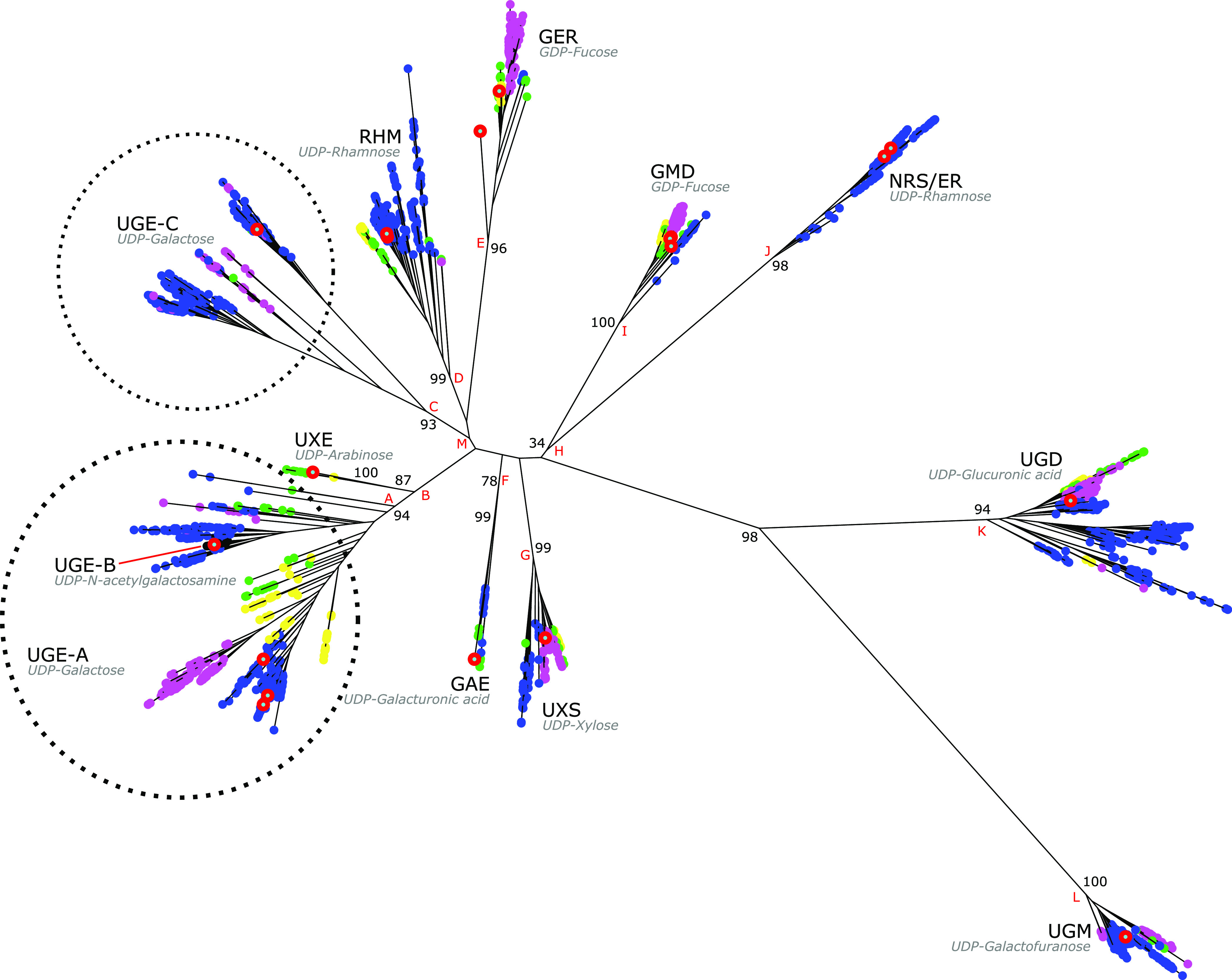
IQ-TREE phylogeny of epimerase/dehydrogenase/dehydratase/mutase nucleotide-sugar interconverting enzymes in 491 fungal taxa. Bootstrap support values are indicated on branches. Nodes are color-coded as follows: blue, Ascomycota; pink, Basidiomycota; green, Blastocladiomycota, Chytridiomycota, and Cryptomycota; yellow, Mucoromycotina, Entomophthoromycotina, Zoopagomycotina, and Kickxellomycotina; orange, Microsporidia; black, UGE-B. A red circle surrounding a node indicates that the corresponding enzymes or enzyme products have been characterized. For clarity in discussion, major nodes are labeled alphabetically.

As shown in Fig. 4, the final IQ-TREE phylogeny for the nucleotide-sugar interconverting epimerase/dehydrogenase/dehydratase/mutase enzymes comprises 10 major clades (splits B, C, D, E, F, G, I, J, K, and L). Biochemically characterized sequences were used to assign clade function as follows: UDP-Glc 4-epimerase (UDP-Glc ↔ UDP-Gal), UDP-GlcNAc 4-epimerase (UDP-GlcNAc → UDP-GalNAc) (UGE-A and UGE-B; split A), UDP-Xyl epimerase (UDP-Xyl ↔ UDP-Ara) (UXE; split B), UDP-galactopyranose mutase (UDP-Gal*p* ↔ UDP-Gal*f*) (UGM; split L), UDP-Glc 6-dehydrogenase (UDP-Glc → UDP-GlcA) (UGD; split K), GDP-Man 4,6-dehydratase (GDP-Man → GDP-Fuc in combination with GER) (GMD; split I), GDP-Fuc synthase (GDP-Man → GDP-Fuc in combination with GMD) (GER; split E), UDP-Xyl synthase (UDP-GlcA → UDP-Xyl) (UXS; split G), UDP-Glc 4,6-dehydratase (UDP-Glc → UDP-Rha in combination with NRS/ER) (RHM; split D), UDP-Glc 4-epimerase (UDP-Glc → UDP-Gal) (UGE-C; split C), UDP-GlcA 4-epimerase (UDP-GlcA ↔ UDP-GalA) (GAE; split F), and nucleotide-rhamnose synthase 3,5-epimerase/4-reductase (UDP-Glc → UDP-Rha in combination with RHM) (NRS/ER; split J) activities (Fig. 2). These data show that the UGE-A, UGE-B, and UGE-C clades do not form a monophyletic grouping: UGE-C clusters with RHM and GER, while UGE-A and UGE-B cluster with UXE.

### Distribution of nucleotide-sugar biosynthetic and interconverting enzymes in sequenced fungi.

**FIG 5:**
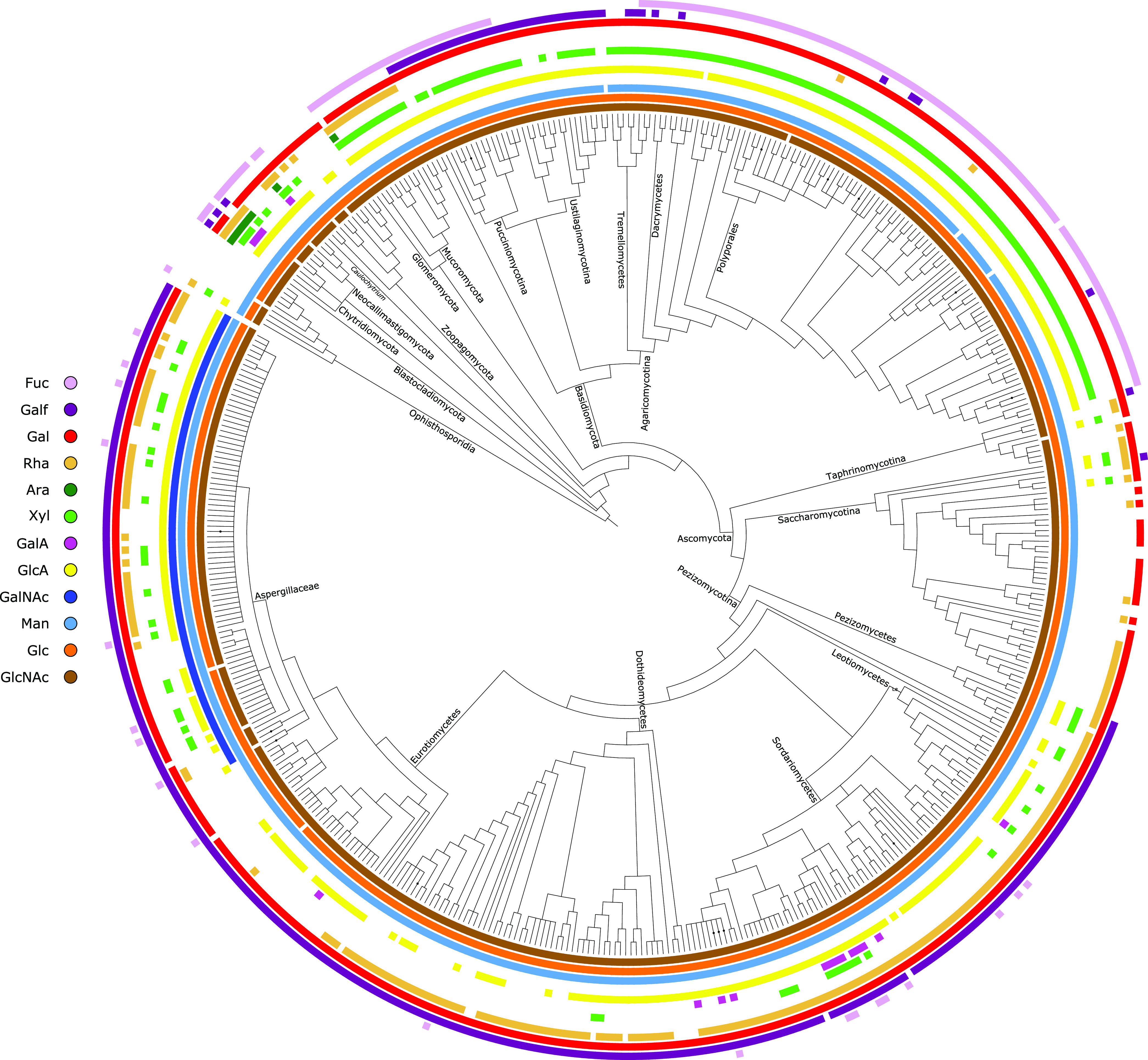
Annotated species tree of 491 fungi. Predicted monosaccharides available in the nucleotide-sugar activated form for incorporation into fungal cell wall glycans are presented as colored concentric rings. Abbreviations: Ara, arabinose; Fuc, fucose; Gal, galactose; GalA, galacturonic acid; GalNAc, *N*-acetylgalactosamine; Gal*f*, galactofuranose; Glc, glucose; GlcA, glucuronic acid; GlcNAc, *N*-acetylglucosamine; Man, mannose; Rha, rhamnose; Xyl, xylose.

**FIG 6:**
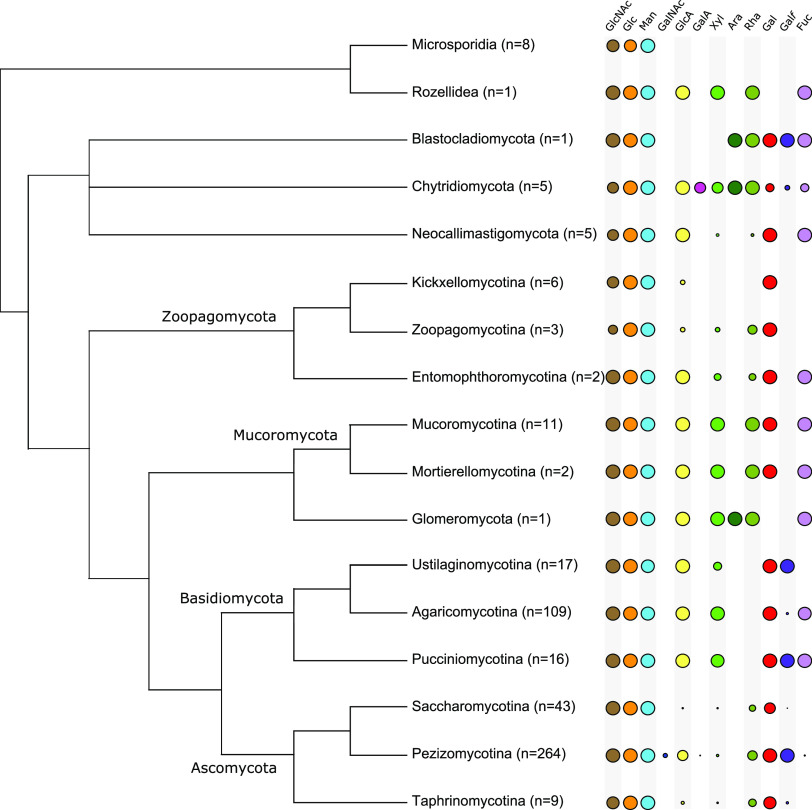
Proportions of species predicted to have specific sugar monomers of glycans within major fungal divisions. Colored circles are scaled to the proportion of species in each taxon predicted to contain a specific sugar. The number of species sampled in each group is given in parentheses after the taxon name. Abbreviations: Ara, arabinose; Fuc, fucose; Gal, galactose; GalA, galacturonic acid; GalNAc, *N*-acetylgalactosamine; Gal*f*, galactofuranose; Glc, glucose; GlcA, glucuronic acid; GlcNAc, *N*-acetylglucosamine; Man, mannose; Rha, rhamnose; Xyl, xylose.

As inferred from the functionally annotated phylogenetic data, the identification of NSI pathways was used to predict the distribution of monosaccharides in the fungi sampled (Fig. 5; see also Fig. S1 in the supplemental material). These results are summarized in Fig. 6, where the prevalence of specific monosaccharides is illustrated for major fungal divisions.

10.1128/mBio.03540-20.2 FIG S1 Annotated species tree of 491 fungi. Predicted monosaccharides available in the nucleotide-sugar activated form for incorporation into fungal cell wall glycans are presented as colored concentric rings. Abbreviations: Ara, arabinose; Fuc, fucose; Gal, galactose; GalA, galacturonic acid; GalNAc, *N*-acetylgalactosamine; Gal*f*, galactofuranose; Glc, glucose; GlcA, glucuronic acid; GlcNAc, *N*-acetylglucosamine; Man, mannose; Rha, rhamnose; Xyl, xylose. Major taxonomic groups are indicated in circles on branches and nodes. Download FIG S1, EPS file, 0.9 MB. Copyright © 2021 Schwerdt et al. 2021 Schwerdt et al. https://creativecommons.org/licenses/by/4.0/ This content is distributed under the terms of the Creative Commons Attribution 4.0 International license.

### Opisthosporidia, Chytridiomycota, Neocallimastigomycota, and Blastocladiomycota.

The Opisthosporidia are basal lineages that deploy cell walls only in sporulating tissues. The phylogenetic results in Fig. 5 and Fig. S1 (summarized in Fig. 6) identify in Microsporidia the enzymatic pathways to synthesize the nucleotide-sugars that carry glucosyl, *N*-acetylglucosaminyl, and mannosyl residues, i.e., the substrates required for the synthesis of the three primary carbohydrate polymers found in all fungi. However, Rozellidea (Opisthosporidia) are shown to be able to synthesize nucleotide-sugars whose monosaccharides are glucosyl, *N*-acetylglucosaminyl, mannosyl, fucosyl, glucurononosyl, rhamnosyl, and xylosyl residues. Completing this group of early-branching, flagellated fungi are the Neocallimastigomycota, Chytridiomycota, and Blastocladiomycota phyla. Chytridiomycota are shown to have the ability to synthesize all sugar donor substrates included in this study with the exception of UDP-GalNAc, i.e., the nucleotide-sugars that carry glucosyl, N-acetylglucosaminyl, mannosyl, fucosyl, galactosyl, galacturonosyl, galactofuranosyl, glucuronosyl, rhamnosyl, and xylosyl residues. Blastocladiomycota present a similar complement but also have lost the capacity to synthesise xylosyl, galacturonosyl and glucuronosyl. Neocallimastigomycota, however, are shown to be able to synthesize only glucuronosyl, rhamnosyl, galactosyl, fucosyl, and xylosyl residues.

### Mucoromycota, Zoopagomycota, and Glomeromycota.

Mucoromycota, Zoopagomycota, and Glomeromycota constitute an unresolved grouping of largely saprotrophic or parasitic hyphal fungi once classified as the phylum Zygomycota. The phylum Zoopagomycota comprises two major subphyla in these data: Kickxellomycotina and Entomophthoromycotina. Kickxellomycotina are shown to have the ability to synthesize nucleotide-sugars whose monosaccharides are glucosyl, *N*-acetylglucosaminyl, mannosyl, galactosyl, and glucuronosyl residues. However, UDP-GlcA has also been lost in the Dimargaritales and Legeriomycetaceae (e.g., the insect symbionts of the genus *Smittium*) (Fig. S1) following the emergence of the Kickxellomycotina. The insect-pathogenic Entomophthoromycotina clade is predicted to synthesize UDP-Glc, UDP-GlcNAc, GDP-Man, UDP-GlcA, UDP-Gal, UDP-Fuc, and, in *Basidiobolus* spp., also UDP-Xyl and UDP-Rha. Finally, these data show that some members of the parasitic and predatory Zoopagales order within Zoopagomycota can biosynthesize UDP-GlcNAc, UDP-Glc, UDP-Man, UDP-GlcA, UDP-Xyl, UDP-Rha, and UDP-Gal. Mucoromycota have the enzyme pathways to synthesize UDP-GlcNAc, UDP-Glc, UDP-Man, UDP-GlcA, UDP-Xyl, UDP-Rha, GDP-Fuc, and UDP-Gal. The Glomeromycota (arbuscular mycorrhizae) are mutualistic symbionts crucial to the ecological viability of plants and are shown to synthesize UDP-GlcNAc, UDP-Glc, UDP-Man, UDP-GlcA, UDP-Xyl, UDP-Rha, GDP-Fuc, and UDP-Ara.

### Ascomycota and Basidiomycota.

The Dikarya are the most speciose of all fungi and account for the majority of species sampled in this work. The majority of Basidiomycota species are predicted to synthesize nucleotide-sugars that carry glucosyl, *N*-acetylglucosaminyl, mannosyl, glucuronosyl, xylosyl, galactosyl, and, with the exception of the plant-parasitic Ustilaginomycotina (true smuts), fucosyl residues. The annotated phylogenetic results also show a consistent presence in the enzyme pathways for the synthesis of UDP-Gal*f* in the Ustilaginomycotina and Pucciniomycotina, with a very sparse distribution across the Tremellales order (jelly fungi), the genus *Dichomitus* (Polyporales; poroid crust fungi), and the polypore genus *Trametes*, and with one species in the genus *Amanita* (mushrooms). UDP-Rha was predicted to be synthesized by Suillus luteus (Boletales) and Phlebia brevispora (Polyporales; white rot).

These data show that the Ascomycota have the ability to synthesize UDP-Glc, UDP-GlcNAc, GDP-Man, UDP-Gal*p*, and, with the exception of the Taphrinales and Saccharomycotina, UDP-Gal*f*. The pathways required for the synthesis of UDP-GlcA are predicted in most lineages, but not in the Taphrinales, *Schizosaccharomyces* (e.g., fission yeast), Saccharomycetales (except for Tortispora caseinolytica, *Lipomyces*, and the oleaginous genus *Yarrowia*), or Tuberaceae (truffles), and are inconsistently distributed in the Dothideomycetes and the Leotiomycetes. The Eurotiomycetes (which include *Penicillium*) have the ability to synthesize UDP-GlcA, with the exception of the Onygenales and some losses in single species. UDP-GalA is shown in the data to be sparsely present in the Ascomycetes, primarily in the entomopathogenic Hypocreales. Similarly, UDP-Xyl is sparsely distributed in the Saccharomycetales, Aspergillaceae, and Pezizomycotina. However, all Pezizomycetes in this study are predicted to be able to synthesize UDP-Xyl, with the exception of the Tuberaceae, and this nucleotide-sugar is present in one species of the Taphrinomycotina (Neolecta irregularis). These data show no ascomycete species predicted to biosynthesize UDP-Ara. The ability to synthesize UDP-Rha is broadly distributed in the Ascomycetes but almost completely lost in the Herpotrichiellaceae and *Penicillium* spp. UDP-Rha is also irregularly distributed in the Aspergillaceae, Dothideomycetes, Saccharomycetales, and early-branching Ascomycota. The ability to synthesize GDP-Fuc is, with the exception of the Taphrinomycotina and Saccharomycetales, widely but sparsely distributed across the Ascomycota.

## DISCUSSION

Innovations of the fungal cell wall have underpinned the spectacular expansion of Fungi that began some 2 billion years ago (22). The cell wall provides the necessary mechanical resistance against environmental pressures and assaults but simultaneously has the flexibility to remodel itself during morphogenesis, to interface with hosts, and to mediate signaling. This structural dynamism is accompanied by significant inter- and intraspecies compositional variations, the extent of which remains to be understood. The present work used phylogenomic analyses of biochemical pathways responsible for the biosynthesis of nucleotide-sugars to elucidate the variation of monosaccharides available for incorporation into cell wall polysaccharides. When considered in an evolutionary context, our results enable inferences of fungal cell wall evolution since the split from metazoans and can be used pragmatically to identify species-specific monosaccharide profiles that can be targeted for the development of antifungal compounds.

The nucleotide-sugar pyrophosphorylases catalyze the conversion of monosaccharide 1-phosphates from the *de novo* and salvage pathways to the primary UDP-sugars used in the biosynthesis of the fungal cell wall polysaccharides: UDP-Glc, UDP-Man, and UDP-GlcNAc. In Fig. 3, experimentally verified enzyme activities that synthesize these nucleotide-sugars cluster with large, well-resolved clades containing all major fungal lineages. This is consistent with the fundamental position of UDP-Glc, GDP-Man, and UDP-GlcNAc in the fungal kingdom and the basal position of these enzyme families. UDP-GlcNAc pyrophosphorylase (UAP) and UDP-Glc pyrophosphorylase (UGP) form single monophyletic groupings; however, mannose-1-phosphate guanylyltransferase (GMPP) comprises three clades. GMPP splits C and D have representatives from all major fungal lineages, implying that an early gene duplication event occurred before any of the early-branching lineages had diverged from the fungal ancestor (Fig. 3, split F). The third clade assigned as GMPP (split E) is Dikarya specific and is separated by a very long branch. This lineage may have been lost in fungal lineages that diversified after the Dikarya divergence, but without data determining branching order, the precise relationship of split E in the GMPP clade requires further analysis. Additionally, GMPP split E does not contain an experimentally characterized enzyme and was assigned a function using protein profile and homology data. Thus, it is possible that functional annotations for this clade require reassessment. Regardless, our evidence that the GMPP gene duplication event occurred early in fungal evolution is consistent with the putative pleiotropic roles of mannose in different fungal metabolic processes (23).

The NSI epimerase/dehydrogenase/dehydratase/mutase phylogeny (Fig. 4) shows an enzyme family that has undergone substantial duplication and neofunctionalization prior to the ancestral split of fungi from the lineage leading to the Metazoa or nucleariid amoebae. Indeed, considering that biochemically characterized sequences from plants and bacteria nest within the UXE, GAE, GER, and GMD fungal clades, this functional differentiation probably occurred prior to the emergence of eukaryotes (24) or through horizontal evolutionary processes. The UGD, UGM, RHM, and UGE-A/B/C clades all comprise basal fungi; however, the NRS/ER clade is resolved to be Dikarya specific. This clade is the only NSI enzyme family in this study that is thought to have evolved following the emergence of the eukaryotes (24). However, the absence of early-branching fungi suggests either missing data, widespread loss in early-branching lineages, or a phylogenetic artifact; the branch leading to the NRS/ER and GMD clades (split H) is poorly supported (34%).

In contrast to the shallow relationships present in other NSI clades, the UGE groups are weakly structured, with relatively deep phylogenetic relationships. As resolved in Fig. 4, the groups are not monophyletic: UGE-C makes up a clade with RHM and GER (split M), whereas UGE-A and -B cluster with UXE (split B). This observation places the UDP-Glc 4-epimerase at the midpoint of the phylogeny and the basal NSI enzyme function. This result is inconsistent with the prevailing model of NSI evolution (24) and inconclusive without further biochemically characterized representatives in UGE-C and well-resolved, deep phylogenetic nodes (see Data Set S2 in the supplemental material). However, the abundance of putative UGE sequences is consistent with the ubiquity of galactose in fungal metabolism (25).

10.1128/mBio.03540-20.3 DATA SET S1 IQ-TREE phylogeny of nucleotide-sugar pyrophosphorylases from 491 fungal taxa in Newick format. Download Data Set S1, TXT file, 0.1 MB. Copyright © 2021 Schwerdt et al. 2021 Schwerdt et al. https://creativecommons.org/licenses/by/4.0/ This content is distributed under the terms of the Creative Commons Attribution 4.0 International license.

10.1128/mBio.03540-20.4 DATA SET S2 IQ-TREE phylogeny of nucleotide-sugar epimerase, dehydrogenase, dehydratase, and mutase enzymes from 491 fungal taxa in Newick format. Download Data Set S2, TXT file, 0.2 MB. Copyright © 2021 Schwerdt et al. 2021 Schwerdt et al. https://creativecommons.org/licenses/by/4.0/ This content is distributed under the terms of the Creative Commons Attribution 4.0 International license.

The weak structure of the UGE-A/B and UXE grouping is evident by the short molecular branch separating them. Similarly, a disassociation between phylogenetic structure and predicted activity exists with UGE-B. The clade comprises sequences of enzymes that are predicted to bind UDP-GlcNAc and catalyze the conversion to UDP-GalNAc. However, as seen in Fig. 4, the ability to interconvert these acetylated substrates evolved subsequent to the emergence of the Aspergillaceae. Taken together, these results resolve UGE as a polyphyletic grouping comprising UGE-A, UGE-B, UGE-C, and UXE. Further work is required to improve confidence in the functional assignments, determine precise class boundaries, and perhaps determine whether convergent evolution, horizontal inheritance, or hitherto undiscovered neofunctionalization has driven the observed phylogenetic structure.

The phylogenetic results provided the opportunity to annotate the presence of specific nucleotide-sugar interconverting and biosynthetic pathways on the fungal species tree. Using these data, we can illuminate macroscale patterns in cell wall evolution in fungi and predict cell wall carbohydrate composition profiles for fungal taxa.

The cytoparasitic Microsporidia are, along with their sister taxon Rozellidea, the most basal lineages in our data. The only nucleotide-sugars predicted in Microsporidia are those synthesized by the UDP-Glc, GDP-Man, and UDP-GlcNAc pyrophosphorylases. This sugar distribution appears consistent with the position of Microsporidia at the base of the fungal tree. However, *Rozella* spp. synthesize four additional nucleotide-sugars (UDP-GlcA, UDP-Rha, UDP-Xyl, and GDP-Fuc), and taxa branching after Microsporidia (Blastocladiomycota and Chytridiomycota) are resolved to synthesize all nucleotide-sugars discussed in this study, with the exception of UDP-GalNAc (Fig. 5 and 6). A loss of these pathways in Microsporidia is probable, because, first, there are ∼60 nucleotide-sugars known in prokaryotes and, second, extreme selection has driven Microsporidia to simplify much of their biology, including their genomes and metabolic pathways (26).

Fungi almost certainly evolved in an aquatic environment (27). Terrestrial colonization had a profound impact on morphology (18), particularly with regard to the advancement of hyphal growth and the loss of flagellated mobility. The Chytridiomycota are primarily flagellated, require water for dispersal, and are relatively speciose in aquatic habitats (27). Along with the Blastocladiomycota, they have rudimentary hyphal growth and diverged from other fungi prior to terrestrial colonization. Subsequent to the emergence of the Mucoromycota, Zoopagomycota, and Glomeromycota (28), we first observe the loss of the flagellum, the expansion of hyphal growth, and a fundamental shift in nuclear organization (19). These clades predominantly occupy terrestrial habitats and are basal to the fungal lineages that expanded on land. As seen in Fig. 4, their diversification corresponds to a significant reduction in the diversity of nucleotide-sugar biosynthetic pathways (Fig. 5 and 6). There is evidence that other carbohydrate enzyme pathways have experienced major gene losses following life strategy transitions. Early fungi are thought to have colonized land before plants, possibly exploiting streptophyte algae, then diversifying as terrestrial lineages expanded. It has been shown that pectinase enzyme families were present in very early fungi, undergoing significant gene loss in lineages that adapted to nonplant eukaryotic hosts (such as arthropods) but duplicating in those that had followed the Embryophytes (29).

This predicted monosaccharide distribution pattern extends into the later-diverging Ascomycetes and Basidiomycetes. In the Dikarya, we observe an irregular distribution of predicted nucleotide-sugar pathways such that resolving the ancestral losses of monosaccharides is impossible. For instance, in the Ascomycota, we identify the pathways for UDP-Rha synthesis throughout the division, with some notable losses in the Eurotiomycetes. In contrast, the majority of Basidiomycota lineages have lost the ability to incorporate rhamnose into their cell walls (Fig. 6). However, we are unable to specify that rhamnose was lost in the Basidiomycetes following the divergence from the Ascomycetes, because two species spread across the division (Phlebia brevispora and Suillus brevipes) and are shown to have RHM and NRS/ER representatives (Fig. S1). Conversely, we observe a near-universal distribution of GDP-Fuc in the Basidiomycetes, with the exception of the Ustilagomycotina, but a very sparse and wide distribution in the Ascomycetes (Fig. 5).

The irregular presence of some monosaccharides in the fungal species tree contrasts with previous observations of a negative association between monosaccharide diversity and fungal expansion. We cannot with confidence specify nodes on the fungal tree where specific nucleotide-sugars have been lost. The scattered presence of some nucleotide-sugars, for example, UDP-Xyl in the Ascomycetes (Fig. 5), suggests that they were lost in taxa subsequent to the emergence of extant fungal species. The selection (or lack thereof) that caused this widespread loss in the current epoch is unknown. Significant gene loss rates have been shown in the Ascomycota, particularly during independent simplification events in yeast lineages subsequent to the evolution of complex multicellularity (30, 31). Whether significant life strategy and phenotypic transitions explain specific nucleotide-sugar losses remains to be determined. Alternatively, other evolutionary mechanisms might explain the fragmented distribution of nucleotide-sugar biosynthesis pathways in the Dikarya. Although still a matter of active debate, horizontal gene transfer (HGT) across species boundaries is thought to be significant to prokaryotic and eukaryotic evolution (32, 33) and has been observed in fungi (34, 35). The trophic strategies of fungi permit the horizontal transfer of genetic material. A scenario where the incorporation of host cell wall enzymes confers an advantage in, for instance, evading the immune response is highly plausible. Such explanations for the predicted irregular distribution of nucleotide-sugars (and therefore monosaccharides) require further investigation.

Variations in genome assembly and annotation completeness may contribute to the inconsistent phylogenetic distribution of monosaccharides. However, we consider this to be unlikely, because the three primary nucleotide-sugars thought to be present in all fungi (UDP-GlcNAc, UDP-Glc, and GDP-Man) are, with a handful of exceptions, resolved in all species. If assembly and annotation quality could completely explain the irregular distribution of monosaccharides, then it is reasonable to expect that we would observe a fragmented presence in the primary nucleotide-sugars also.

This work has made explicit the range of monosaccharides in specific fungal taxa. In particular, xylosyl residues have been biochemically shown to be present in Cryptococcus neoformans, and homology data have suggested a wider distribution (36), but these data show significant losses in Ascomycota, the most speciose fungal lineage. Fucose has been chemically shown to occur in the Mucoromycota (6, 37, 38), but although previous phylogenetic observations predicted fucose in the Basidiomycetes (39), we reveal here the scattered presence of fucose in Ascomycota. Galactofuranose is predicted both here and by Tefsen et al. (40) to be widespread across the Ascomycota. Additionally, we resolve a few Basidiomycetes that are able to synthesize this nucleotide-sugar—the inverse distribution to that observed for fucose and xylose. Such patterns of predicted monosaccharide distribution inform the development of antifungal compounds and illustrate the dynamic and varied evolutionary history of the fungal cell wall.

## MATERIALS AND METHODS

### Initial sequence data acquisition.

Representative sequences from the *de novo* nucleotide-sugar biosynthetic and interconverting pathways were accessed from the Kyoto Encyclopedia of Genes and Genomes (KEGG) database (https://www.genome.jp/kegg-bin/show_pathway?map00520/cpd:C00029/cpd:C00103) (41) using Enzyme Commission (EC) numbers corresponding to relevant nodes in the network. Selected EC numbers were used to search the DOE Joint Genome Institute (JGI) MycoCosm database (https://genome.jgi.doe.gov/programs/fungi/index.jsf) (42) for additional protein sequence data from publicly available fully sequenced genomes.

### Quality control of the initial sequence data.

Sequences for each EC number were aligned using MUSCLE (43), and phylogenies were built with FastTree (version 2.1.5) (44) using the Jones-Taylor-Thornton (JTT) substitution model (45) and 20 gamma rate categories. To investigate putative protein functions and confirm the protein sequences of the initial data, a BLAST (BLASTP) (46) search of each protein sequence was performed against the NCBI nonredundant (nr) and Swiss-Prot databases. Additional annotation data were obtained from local InterProScan searches (47) to assign putative functions. Finally, experimentally characterized fungal and bacterial sequences from major identified nucleotide-sugar interconverting enzymes were included as functional references (Table S1, Data Set S1, and Data Set S2). Based on the multiple sequence alignment, phylogeny, homology, and annotation results, obvious outliers were discarded from subsequent analyses.

10.1128/mBio.03540-20.1 TABLE S1 List of experimentally characterized fungal and bacterial sequences from major nucleotide-sugar interconverting enzymes included as functional references in this study. Download Table S1, PDF file, 0.05 MB. Copyright © 2021 Schwerdt et al. 2021 Schwerdt et al. https://creativecommons.org/licenses/by/4.0/ This content is distributed under the terms of the Creative Commons Attribution 4.0 International license.

### Protein profile searches of available fungal genomes.

To characterize *de novo* nucleotide-sugar biosynthetic and interconverting pathways in available fungal genomes, hmmbuild (48) was used to construct hidden Markov models (HMM) (48) for each NSI EC category using the curated alignments. Since no candidate sequences existed in either the KEGG or the JGI database for EC 5.1.3.-, representing the UDP-Rha synthase 3,5-epimerase/4-reductase (NRS/ER) enzyme family, the HMM was constructed using data from Martinez et al. (17). Hmmsearch (48) was used to search the JGI MycoCosm database for matches to the constructed NSI profiles, and specific NSI class E value thresholds were determined through inspection of InterProScan, BLAST, and i-TASSER (49) results.

### Phylogenetic analysis and identification of sequence motifs specific to each enzyme.

The AlignSeqs function from DECIPHER (50) was used to align the final curated protein sequences for each NSI EC class. The NSI enzymes included in this study encompass two major classes, the pyrophosphorylases and epimerases/dehydrogenases/isomerases. Multiple sequence alignments of these superfamilies were also constructed using AlignSeqs with the default parameters.

IQ-TREE was used to infer phylogenetic trees for both the pyrophosphorylase and epimerase/dehydrogenase/isomerase superfamilies. Model selection was performed using the integrated ModelFinder (51) with WAG+R10 as the best-fit model for both data sets. IQ-TREE was run with a maximum of 1,000 ultrafast bootstrap replicates and nearest-neighbor interchange optimization (-bnni). For each data set, 15 independent tree inferences were performed, and the highest-likelihood iteration was selected as final.

### Prediction of the presence or absence of each nucleotide-sugar on the fungal species tree.

A fungal species tree was constructed using iTOL (https://itol.embl.de/) (52) with data from the NCBI taxonomy database (53). The tree was modified to correspond to recent literature (18, 54–73).

### Data availability.

The HMM files used in this work have been deposited at FigShare (https://doi.org/10.25909/14091062).
