# Supplementary material for: Phylogenomic Analyses of Nucleotide-Sugar Biosynthetic and Interconverting Enzymes Illuminate Cell Wall Composition in Fungi
Source: mBio. 2021 Apr 13;12(2):e03540-20. doi: 10.1128/mBio.03540-20 (PMC8092308; doi:10.1128/mBio.03540-20)
Supplement: TABLE S1 [file mBio.03540-20-st001.pdf]

Sequence accession number	*Phylum*	*Species*	*Enzymes*		*Reference*
AAL07003.1	Plant	*Arabidopsis thaliana*	GAE	UDP-glucuronate 4-epimerase	(Usadel *et al.*, 2004)
AAK95561.1	Fungi	*Cryptococcus neoformans*	GDH	UDP-Glucose 6-dehydrogenase	(Maor *et al.*, 2004)
AAC77843.1	Bacteria	*Escherichia coli*	GER	GDP-Fucose synthase	(Mattila *et al.*, 2000)
ADC54121.1	Fungi	*Mortierella alpina*	GER	GDP-Fucose synthase	(Ren *et al.*, 2010)
AAC77842.1	Bacteria	*Escherichia coli*	GMD	GDP-Mannose 4,6-dehydratase	(Mattila *et al.*, 2000)
ANT95329.1	Fungi	*Mortierella alpina*	GMD	GDP-Mannose 4,6-dehydratase	(Ren *et al.*, 2010; Wang *et al.*, 2016)
ADC54120.1	Fungi	*Mortierella alpina*	GMD	GDP-Mannose 4,6-dehydratase	(Ren *et al.*, 2010; Wang *et al.*, 2016)
AAY40351.1	Fungi	*Aspergillus fumigatus*	GMPP	Mannose-1-phosphate guanylyltransferase	(Jiang *et al.*, 2008)
AEH41994.1	Fungi	*Magnaporthe oryzae*	NRS/ER	3,5-epimerase/4-reductase	(Martinez *et al.*, 2012)
AEH41996.1	Fungi	*Botrytis cinerea*	NRS/ER	3,5-epimerase/4-reductase	(Martinez *et al.*, 2012)
AEH41993.1	Fungi	*Magnaporthe oryzae*	RHM	UDP-Glucose 4,6-dehydratase	(Martinez *et al.*, 2012)
AEH41995.1	Fungi	*Botrytis cinerea*	RHM	UDP-Glucose 4,6-dehydratase	(Stewart & Copeland, 1998)
DAA11757.1	Fungi	*Saccharomyces cerevisiae*	UAP	UDP-N-acetylglucosamine pyrophosphorylase	(Milewski *et al.*, 2006)
AOW29678.1	Fungi	*Candida albicans*	UAP	UDP-N-acetylglucosamine pyrophosphorylase	(Milewski *et al.*, 2006)
XP_754823.1	Fungi	*Aspergillus fumigatus*	UGE	UDP-Glucose 4-epimerase (UDP-N-acetylglucosamine 4-epimerase)	(Lee *et al.*, 2014)
XP_746467.1	Fungi	*Aspergillus fumigatus*	UGE	UDP-Glucose 4-epimerase (UDP-N-acetylglucosamine 4-epimerase)	(Lee *et al.*, 2014)
XP_753568.1	Fungi	*Aspergillus fumigatus*	UGE	UDP-Glucose 4-epimerase (UDP-N-acetylglucosamine 4-epimerase)	(Lee *et al.*, 2014)
CBF76914.1	Fungi	*Aspergillus nidulans*	UGE	UDP-Glucose 4-epimerase (UDP-N-acetylglucosamine 4-epimerase)	(El-Ganiny *et al.*, 2010)
NP_009575.1	Fungi	*Saccharomyces cerevisiae*	UGE	UDP-Glucose 4-epimerase (UDP-N-acetylglucosamine 4-epimerase)	(Majumdar *et al.*, 2004)
CAI38754.2	Fungi	*Aspergillus fumigatus*	UGM	UDP-Galactopyranose mutase	(Bakker *et al.*, 2005; Schmalhorst *et al.*, 2008; Oppenheimer *et al.*, 2010)
CAA81872.1	Fungi	*Saccharomyces cerevisiae*	UGP	UDP-Glucose pyrophosphorylase	(Daran *et al.*, 1995)
ADK79128.1	Bacteria	*Sinorhizobium meliloti*	UXE	UDP-Xylose epimerase	(Gu *et al.*, 2011)
AAK59981.1	Fungi	*Cryptococcus neoformans*	UXS	UDP-glucuronate decarboxylase/UDP-Xylose synthase	(Bar-Peled *et al.*, 2001)

Supplementary Table 1
